# Numerical synesthesia is more than just a symbol-induced phenomenon

**DOI:** 10.3389/fpsyg.2013.00860

**Published:** 2013-11-14

**Authors:** Limor Gertner, Isabel Arend, Avishai Henik

**Affiliations:** Department of Psychology and the Zlotowski Center for Neuroscience, Ben-Gurion University of the NegevBeer-Sheva, Israel

**Keywords:** number-color synesthesia, number-space synesthesia, symbolic numbers, non-symbolic magnitude comparison, numerical cognition

Synesthesia is a peculiar condition that involves an atypical binding between two seemingly independent sensory modalities or within the same one. All synesthetic bindings are characterized by an inducing stimulus (i.e., inducer) and a subjective sensation triggered concurrently (i.e., concurrent). Synesthetic inducers can be sensory (e.g., sound) or conceptual (e.g., graphemes, time units) while the concurrent sensation is, in the majority of cases, a sensory one (e.g., smell, touch).

In numerical synesthesia, numbers (i.e., inducer) automatically and consistently trigger an ancillary experience of color, texture, spatial location, or personification (i.e., concurrent). For example, for a given synesthete, an audition of the number 5 may trigger a sensation of the color shiny yellow, be mapped on a vertical meridian above four and beneath six in his/her peripersonal space or elicit a cognitive awareness of “a young man, ordinary, and common in his tastes and appearance …” (Simner et al., 2011).

Until recently, numerical synesthesia was almost unquestionably viewed as a symbolic-based phenomenon. This was mainly because most synesthetes report their synesthetic experience is elicited solely by symbolic content (i.e., Arabic numbers) but not by non-symbolic ones (i.e., size, quantity) (Cohen Kadosh and Gertner, 2011). Furthermore, some researchers showed that non-symbolic magnitudes (i.e., random clusters of dots) or less familiar symbolic numerals (i.e., Roman numbers) were ineffective in evoking the synesthetic concurrent (Ramachandran and Hubbard, 2001, Experiment 3; Ward and Sagiv, 2007), suggesting that Arabic numbers *per se* (i.e., their form, ordinality, etc.) and not their semantic meaning or numerosity are the critical factors for inducing synesthetic experience (Hubbard et al., 2009).

However, when considering two main findings in the domain of numerical cognition—(a) that symbolic content is intimately associated with non-symbolic dimensions (e.g., size, quantity, brightness) (e.g., Schwarz and Heinze, 1998; Fias et al., 2003; Pinel et al., 2004; Ansari, 2008; Cohen Kadosh et al., 2008a), and (b) that magnitude is assumed to be automatically activated whenever we are presented with numbers (Dehaene et al., 1993; Dehaene and Akhavein, 1995)—one must wonder whether synesthetic experience can also be elicited by different magnitude dimensions.

In this opinion paper we present some recent observations from the literature on numerical synesthesia indicating that magnitude representation may play a role in mediating synesthetic effects found under experimental settings. Based on this evidence we suggest that synesthetic experiences induced by numbers may be produced also by non-symbolic magnitudes, due to the cognitive and neuronal overlap of these two dimensions. In other words, we propose that numerical synesthesia is more than a symbol-induced phenomenon *per se*. Furthermore, we speculate that this suggested association between a non-symbolic inducer and a synesthetic concurrent may manifest at different levels of awareness, resulting in an explicit, reportable experience for some synesthetes but a more non-conscious or implicit representation in others.

Before presenting our arguments, it is important to note that we use the phrase “numerical synesthesia” to include the subtypes of synesthesia that share numerical inducers (i.e., number-color and number-space synesthesia). We acknowledge that in spite of a common inducer (i.e., number), different mechanisms may mediate the various inducer-concurrent associations (Novich et al., 2011), yet we argue that both types discussed here illustrate the suggested involvement of magnitude representation in inducing synesthetic concurrents (i.e., color, spatial location).

## Number-color synesthesia

Berteletti et al. (2010) tested a grapheme-color synesthete (NM) who reported experiencing colors in response to digits, but not in response to non-symbolic numerosities. Surprisingly, when this synesthete was tested on a Stroop-like color naming task, he showed a congruency effect for both Arabic digits and dot patterns. The authors suggested that NM had an explicit association of numbers and colors (i.e., primary synesthetic connection) and an implicit association of numerosity and colors (i.e., secondary synesthetic connection). According to the authors, this implicit association, which they refer to as “*pseudosynesthesia*,” is a consequence of an inherent lifetime association between numbers, their magnitudes, and “their” synesthetic colors.

The above study exemplifies an implicit association between *discrete* numerosity and color. However, since non-symbolic discrete numerosities can be counted and labeled by a symbolic number, we cannot affirm that the Stroop-like effect that was found indeed represented a magnitude-based association.

A couple of studies, although it was not their main goal, demonstrated a potential existence of implicit associations between non-symbolic *continuous* magnitudes and colors. Cohen Kadosh and Henik (2006) tested a digit-color synesthete (IS) on an adjusted Stroop-like task with colored lines. The synesthete's task was to decide which of two presented lines was physically longer. Lines were colored either congruently (i.e., a long line presented in a color that was induced by a large digit like 7) or incongruently (i.e., a longer line presented in a color that was induced by a small digit like 2). Digits were not presented at any stage. A congruency effect between line length and color was observed. In a later study, Cohen Kadosh et al. (2007) showed similar effects when they examined the brain activity of the same synesthete while he performed symbolic (i.e., Arabic numbers) and non-symbolic (i.e., triangle height) magnitude comparison in a Stroop-like task similar to the one they used in their previous study (Cohen Kadosh and Henik, 2006). A behavioral congruency effect was observed for physical comparisons for the synesthete but not for controls. Importantly, this congruency effect was found to modulate the event-related potential (ERP) wave N170, suggesting a detection of a conflict between size and color^1^. The above results were interpreted by the authors as an indication for bidirectionality in synesthesia (i.e., colors evoke numbers).

Bidirectionality is an intriguing topic on its own, and the above studies present convincing data supporting it. However, we would like to further interpret these results as suggestive that, for some synesthetes, magnitude might become associated with color (in the absence of symbolic number).

Specifically, we suggest that the congruency effects found between magnitude and color in the studies presented above may be driven by two different conflicts: bidirectionality is responsible for one—that is, the conflict between one physical dimension of the stimuli (size) and the *associated feature* (Arabic number) of the other physical dimension of the stimuli (color). The second conflict, which we believe is more primary, is a conflict between the two physical dimensions of the presented stimuli (size and color). We have several reasons to believe that the latter conflict (i.e., between size and color) happens prior to the former conflict (between size and the *associated* number). First, processing size is faster and easier than processing numbers (Tzelgov et al., 1992; Leibovich and Henik, 2013). Second, while the conflict between the two physical dimensions (i.e., primary conflict) involves only one process—comparing magnitude and color—the conflict between the physical and associated dimension (i.e., secondary conflict) involves two processes—activation of the number by the color (i.e., bidirectionality) and comparing the symbolic and non-symbolic magnitude dimensions (i.e., number and size).

To sum up, we suggest that the congruency effects found for the non-symbolic content in these studies may also be a consequence of conflict between the magnitude of a stimulus and the magnitude associated with the stimulus color, which arises via the implicit interaction between the color and magnitude dimensions. This assumed magnitude-color association is less accessible to the beholder, whether it is a researcher who examines the data or a synesthete who introspects his/her experience, since typically it is covered by the synesthetes' explicit symbolic number-color association. However, the data is currently insufficient for determining conclusively whether a direct magnitude-concurrent conflict exists and if it does, whether it happens earlier than the magnitude-number conflict.

## Number-space synesthesia

The above suggestion is further supported by studies in the area of number-space synesthesia. Recently, Gertner et al. (2012) tested two number-space synesthetes and a group of controls on a simple comparison task with three different types of stimuli: (a) Arabic numbers (symbolic stimuli), (b) patterns of dots (non-symbolic discrete stimuli), and (c) sizes of squares (non-symbolic continuous stimuli). Congruency between magnitude and spatial location was manipulated according to the synesthetic number-space association. The authors found that synesthetes showed a magnitude–space compatibility effect for dot clusters as well as for Arabic numbers. That is, synesthetes were slower to compare two arrays of dots when these non-symbolic magnitudes were presented incompatibly with their symbolic number-space representation. Such a magnitude–space association was not found for controls. In this study, the authors managed to find a magnitude-space congruency effect only for discrete magnitudes but not for continuous magnitudes (physical size). These findings cannot entirely rule out the possibility that the dot patterns activated a symbolic number, which in turn, induced the spatial concurrent. However, in a later study of Gertner et al. (2013), a magnitude-space association was observed also for continuous physical sizes. In that study, two groups of synesthetes (and matched controls) performed a numerical Stroop-like task (Henik and Tzelgov, 1982). One synesthetic group visualized numbers in a rightward orientation (left to right) and the other group visualized numbers in an upward orientation (bottom to top). Participants had to decide which number was physically or numerically larger (in different blocks). An interesting finding was observed in the neutral condition of the physical block (e.g., 3 3) in which numerical value was kept constant but the stimuli location on the screen and physical size varied^2^. Synesthetes of both groups, but not controls, showed a size-space interference effect. For example, for a synesthete with an upward number representation, a smaller 3 at the top and a bigger 3 at the bottom were harder to compare (longer RTs) than vice versa. Similarly, for a synesthete with a rightward representation, a smaller 3 on the right and a bigger 3 on the left was harder to compare than vice versa. The authors suggested that physical size is associated with spatial location according to the directionality that corresponds to the synesthetic symbolic number-form. These findings reflect an association that incorporated the non-symbolic dimension and the spatial concurrent—an association that we assume is inherent in the link between numbers and space in number–space synesthesia.

Taken together, the data presented above suggest that synesthetic association is not only restricted to concrete symbolic inducers but can also be evoked by non-symbolic, discrete, and continuous information. However, it is not clear to what extent synesthetes are aware of such synesthetic associations (and thus, they may be referred to only as semi-synesthetic associations). The data we have at present are still too premature to address this query. However, it seems that there are individual differences regarding this matter. We have noticed over the past several years that some synesthetes do explicitly report having colors or a spatial arrangement for non-symbolic discrete stimuli (i.e., conscious experience of the association); some synesthetes notice it only during a task, after which they report that the incongruent condition was harder or uncomfortable for them (i.e., possibly a semi-conscious experience); and other synesthetes do not report any synesthetic experience in response to non-symbolic stimuli, however, their results indicate such an association (i.e., non-conscious process). Unfortunately, we do not have reliable statistical data about the proportion of synesthetes who explicitly report having a synesthetic association for non-symbolic stimuli.

**Figure 1:**
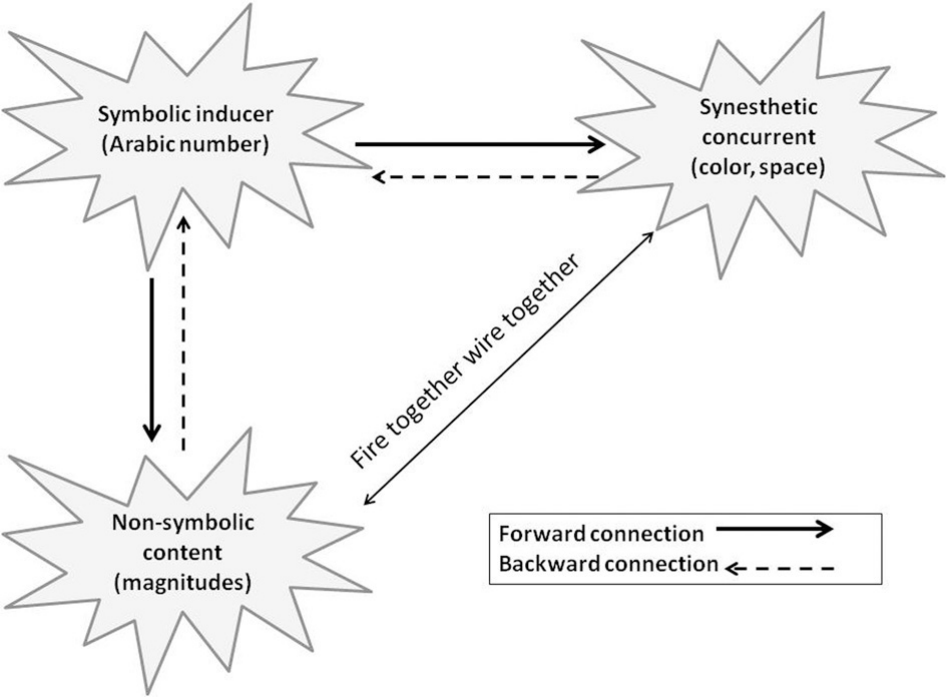
**For numerical synesthetes, symbolic numbers automatically activate both number magnitude (and other non-symbolic content) and their specific concurrents.** The activated entities fire back to the inducer. This simultaneous feedback to the same target forms an autonomous connection between the synesthetic concurrent and the non-symbolic content. However, this connection is not always strong enough to cross the consciousness threshold; thus, in most cases it remains implicit.

How does this magnitude-based semi-synesthetic association arise? Magnitude is a property inherent in symbolic numbers. In the case of synesthesia, symbolic numbers have the capability of inducing the experience of color or space. Thus, after endless “trials” of symbolic inducer-concurrent associations, it is reasonable that the magnitudinal content adopts some capability to autonomously induce a similar synesthetic concurrent. Specifically, when synesthetes encounter a numerical inducer, the synesthetic concurrent and number magnitude are automatically triggered (Cohen Kadosh et al., 2008b). In turn, this semantically related content (magnitude, space, color, etc.) “fires” back, co-activating the inducer. This repeated backward activation forms autonomous connections between the concurrent and the non-symbolic content of the inducer [based on the rationale of Hebb's (1949) axiom—neurons that fire together wire together] (see Figure 1). Thus, non-symbolic properties that are functionally and anatomically attached to the symbolic numerical system become inevitably associated with the synesthetic concurrent, creating magnitude-based synesthetic associations.

However, in some cases these connections are not strong enough to cross the threshold for a conscious experience, thus, resulting in an implicit, rather than explicit, association between non-symbolic numerical information and colors or space.

## Summary

Numbers cannot be disentangled from their semantic meaning. For numerical synesthetes, numbers also cannot be disentangled from their color, space or personality profile. Thus, with time, connections between numbers' semantic networks and synesthetic concurrents are obligatorily formed. In this paper we presented some introductory evidence for this idea; however, further research is needed in order to systematically test the potential existence of implicit (and explicit), direct or indirect associations between non-symbolic continuous magnitude information like size, length, brightness, etc., and synesthetic concurrents. Such research can further enrich our knowledge about the semantic network of numbers and the shared mechanisms of numbers and magnitudes.

## Footnotes

^1^Please note that we are referring only to the finding regarding the non-symbolic comparison.

^2^There were many findings in this research but we are referring only to the one that is relevant for the current opinion paper.
